# Ethyl lactate production by reactive distillation – optimization of reaction kinetics and energy efficiency

**DOI:** 10.12688/openreseurope.13744.1

**Published:** 2021-07-20

**Authors:** Peter Stipsitz, Michael Mandl, Michael Harasek

**Affiliations:** 1 tbw research GmbH, Grünbergstrasse 15, Vienna, 1120, Austria; 2 Institute of Chemical, Environmental and Bioscience Engineering, TU Wien, Getreidemarkt 9/166, Vienna, 1060, Austria

**Keywords:** ethyl lactate, reactive distillation, process intensification, biorefinery

## Abstract

**Background: **Ethyl lactate is an environmentally benign solvent, which could substitute petrol-based volatile organic compounds (VOCs) in many applications if production costs are reduced. It is usually produced by the esterification of lactic acid with ethanol – two important chemical building blocks of biorefineries that are available at industrial scale. Reactive distillation is a promising alternative production process, which utilises process intensification to increase energy efficiency and space-time yield by enhancing the reaction kinetics.

**Methods: **In this work, process intensification of ethyl lactate production by means of distillation was analysed with special focus on the efficient separation of water. The feedstock requirements were studied and the process was optimized regarding reaction kinetics in experiments on laboratory level. The preparation of anhydrous starting mixtures for ethyl lactate formation was tested in batch experiments and applied to reactive distillation. The simultaneous distillation was optimized to ensure that the by-product water was separated efficiently and the separation capacity was not limiting the reaction rate. Combined reactive distillation was compared to a serial setup of reactors and distillation steps.

**Results: **It was found that an optimized serial setup can offer similar process intensification at a lower distillate rate compared to simultaneous reactive distillation.

**Conclusions: **The serial setup is more flexible and straight-forward to regulate and scale-up. Based on the experimental results a continuous production process that uses process intensification to reach high ethyl lactate yield and purity was proposed.

## Plain language summary

Ethyl lactate is an environmentally-friendly and safe chemical, which is produced from biological resources. It could replace conventional petrol-based solvents in many products such as paints and cleaning agents, which could improve air quality and contribute to the reduction of carbon dioxide (CO _2_) emissions. However, the production cost of ethyl lactate has to be reduced for these applications. In this article we present the research we did into the development of an alternative process for the production of ethyl lactate from ethanol and lactic acid, which is called reactive distillation. It is an efficient process that could save energy and equipment costs. We investigated the influence of feedstock composition and different technical process parameters in laboratory experiments. It was important to reduce the water content of the feedstock to achieve good performance results. We concluded that a series of process steps is more efficient than an integrated design in a single apparatus. Based on these results process design for a larger production scale is being continued.

## Introduction

Ethyl lactate is an environmentally benign solvent, which is typically produced from bio-based feedstock. It exhibits very low toxicity, is not ozone-depleting and is readily bio-degradable ( Pereira *et al*., 2011). Ethyl lactate shows favourable solvency properties for many applications. It has the potential to substitute petrol-based volatile organic compounds (VOCs) in a vast range of applications if production costs are reduced ( de Jong *et al.*, 2010). Ethyl lactate is especially interesting for applications that include the release of solvent into the environment, such as coatings, paints and domestic cleaning products. According to the EU emission inventory report, domestic solvent use and coating applications together accounted for 27% of the total non-methane volatile organic compound (NMVOC) emissions in the EU in 2017 ( European Environment Agency, 2019). Substituting conventional solvents by ethyl lactate could offer health and environmental benefits.

**Figure 1.**
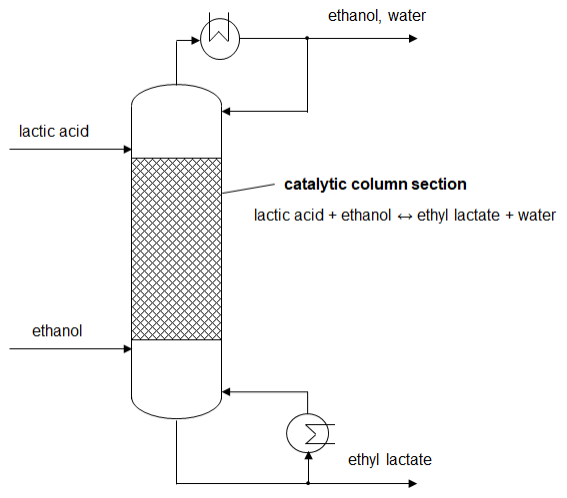
Schematic illustration of ethyl lactate production by reactive distillation.

Ethyl lactate is usually produced by the esterification of lactic acid with ethanol using an acid catalyst. Both reactants are important building blocks of biorefineries that are available at industrial scale. Production processes for ethyl lactate have been patented ( Martino-Gauchi & Teissier, 2004; Martino-Gauchi & Teissier, 2007; Tretjak *et al.*, 2006; Tretjak & Teissier, 2004) and industrialized ( Rosales-Calderon & Arantes, 2019). However, these processes are characterized by low reaction rates and consequently, high capital and operating costs. A number of alternative processes are being developed, the most promising being membrane processes and reactive distillation (see Figure 1) ( Pereira *et al.*, 2011). These processes utilize the effect of process intensification.

By removing the side product water from the mixture during reaction, the equilibrium limitation of conventional batch reactors is overcome. The chemical equilibrium gets re-established following the law of mass action. Therefore, lactic acid conversion above the batch equilibrium can be reached. The driving force is retained at a high level and the reaction kinetics is enhanced. ( Keller, 2014) As a result, the energy efficiency and space-time yield of such processes are considerably higher.

The production of ethyl lactate by reactive distillation has been previously studied by Asthana *et al.* (2005), who tested the simultaneous esterification and separation of ethanol and water in a single apparatus and reached a lactic acid conversion of >90% and a ethyl lactate yield of 73%, but accepted high amounts of oligomers in the raw product stream, which required an additional process unit for refining. Gao *et al.* (2007) optimized operation parameters and reported an ethyl lactate yield of 53%. In the presented work the process was optimized regarding reaction kinetics by experiments at laboratory level and assessed in comparison to a serial setup of reactors and distillation units. Special focus lay on the feedstock requirements and the efficient separation of water. Calculations regarding material and energy balances were added. Based on the results a continuous production process that uses distillation for process intensification was proposed. The described actions were taken in preparation for piloting.

A heterogeneous catalyst was used to enable easy separation. Amberlyst resins were chosen, because they show favourable catalytic properties at low water concentrations ( Nguyen *et al.*, 2018) and are commonly used in literature ( Pereira *et al.*, 2011). Three different cation-exchange resins were tested in batch experiments.

Delgado *et al.* (2007) found that the positive effect of excess ethanol on the equilibrium constant is declining. A molar reactant ratio of n _EtOH_/n _LA_= 3/1 was proposed as optimal value in literature ( Asthana *et al.*, 2005), according to which the starting mixtures of all experiments in this work were prepared.

Technical grade input chemicals (especially lactic acid feedstock) contain high amounts of water, which is undesired in the process. As side-product of the esterification reaction, water limits reaction rate and lactic acid conversion. The reaction kinetics of the esterification of lactic acid with ethanol has been studied extensively, using different lactic acid concentrations from dilute (approx. 20 w%) to concentrated lactic acid (88 w%). Pereira *et al.* (2011) gave a comprehensive review of kinetics studies literature. However, the concentration range above commercially available 88 w% lactic acid solutions has not yet been considered.

Due to its bifunctional structure, lactic acid undergoes intermolecular self-esterification and tends to form oligomers in concentrated solutions ( Asthana *et al.*, 2005). For example, in technical (88 w%) lactic acid solutions at equilibrium, 33% of lactic acid equivalents are present in the form of oligomers ( Vu *et al.*, 2005). However, oligomerization is supressed by the presence of ethanol. If a technical grade (88 w%) lactic acid solution is mixed with ethanol at a molar ratio n _EtOH_/n _LA_ =1, the oligomer content at equilibrium is reduced to 2.4 mol%, and at a ratio of n _EtOH_/n _LA_ =3 it is 0.4 mol%.( Pereira *et al.*, 2008) The feasibility of ethyl lactate formation with dehydrated feedstock was first tested in batch experiments and then applied to reactive distillation.

The production of ethyl lactate is not a straight-forward reactive distillation task, where one of the reaction products exhibits the lowest or highest volatility of the quaternary mixture. Total acid conversion and the recovery of pure ester product cannot be easily achieved by optimization of column operation conditions ( Asthana *et al.*, 2005). Using a distillation column with a reflux ration RR>0 in the case of ethyl lactate formation would concentrate ethanol in the distillate and consequently, retain water in the system. In this work, kinetics was evaluated in a setup for semi-batch reactive distillation without a column. An ethanol feed was employed as a stripping agent for water. The target was to reach close to entire lactic acid conversion and ethyl lactate yield, while keeping losses by oligomerisation as low as possible.

It is expected that the capacity for water removal increases with purity of the ethanol feed. Asthana *et al.* (2005) suggested that an azeotropic ethanol-water feed mixture could be used to produce ethyl lactate via reactive distillation, which would have significant economic advantages. In this work the influence of ethanol feed purity was tested in reactive distillation experiments on laboratory level.

In reactive distillation, where the heterogeneously catalysed esterification reaction is combined with a simultaneous separation operation, several effects can act limiting to lactic acid conversion:

• The chemical reaction at the catalytic site – characterized by the concentration of the components and the catalytic capacity of the catalyst particles.
• Mass transport between catalyst and bulk phase – For Amberlyst resins this step can be neglected at moderate stirrer speed ( Chakrabarti & Sharma, 1993; Delgado *et al.*, 2007).
• Distillation: The separation of water from the bulk phase can potentially be the rate-determining step. To determine the optimal operation conditions, a variation of the stripping agent (ethanol feed purity) and distillate rate was performed.

## Methods

### Materials

Lactic acid (88 w%) was purchased from Carl Roth (Austria), ethanol of two purity levels (96% and 99.9%) was purchased from Australco (Austria). The cation-exchange resins Amberlyst 15, Amberlyst 46 and Amberlyst 48 (Dow, Germany) were used as acid catalysts.

### Analytics

Samples of the reaction mixture of 2 mL volume were cooled to ambient temperature immediately and stored at approx. 281 K for analytical measurements. Lactic acid, ethanol and ethyl lactate were quantified by HPLC measurements. A Bio-Rad Aminex HPX-87H column (300 mm × 7.8 mm) was used with 5 mM sulfuric acid (aqueous solution) as mobile phase. Lactic acid and ethyl lactate were detected with an UV sensor (Agilent G1315B at 224 nm), ethanol was detected by a refractive index sensor (Agilent G1362A). The composition of binary mixtures of ethanol and water (distillate) was determined by density measurements using an oscillating U-tube device (DMA 35, Parr).

### Experimental procedures

***Batch experiments.*** The evaluation of different catalysts and starting mixtures was done in batch experiments. Ethanol and lactic acid were mixed thoroughly in a 0.25 L round bottom flask equipped with a reflux condenser at an initial molar ratio of n _EtOH_/n _LA_ = 3 (0.17 kg reaction mixture). The mixture was heated to reflux temperature (355 K) using a thermal oil bath. A reference sample was taken as a starting point before the catalyst was added to the mixture (weight fraction w _cat_ = 0.1). Three different cation-exchange resins were tested as catalysts for ethyl lactate formation using commercial grade lactic acid (88 w%) and ethanol (96%). Amberlyst 46 showed the highest catalytic activity under the studied conditions and was used in all following experiments.

The influence of the initial water content on the esterification reaction was studied using different starting mixtures as listed in Table 1. For batch No. 1 an 80 w% lactic acid solution was provided by adding deionized water to a commercial lactic acid solution. In batch No.2 the technical grade lactic acid solution was used directly. For batch No.3 and No.4 highly concentrated lactic acid solutions were prepared by a pre-treatment via vacuum distillation at 2 kPa and 353 K using a rotary evaporator (R205, Büchi).

**Table 1.** Starting mixtures for batch kinetics experiments.

Batch No.	Ethanol purity (vol%)	Lactic acid purity (w%)	Initial water fraction (mol/mol)
1	96	80	0.29
2	96	88	0.19
3	96	100 (pre-treated)	0.09
4	99.9	100 (pre-treated)	0.00

**Figure 2.**
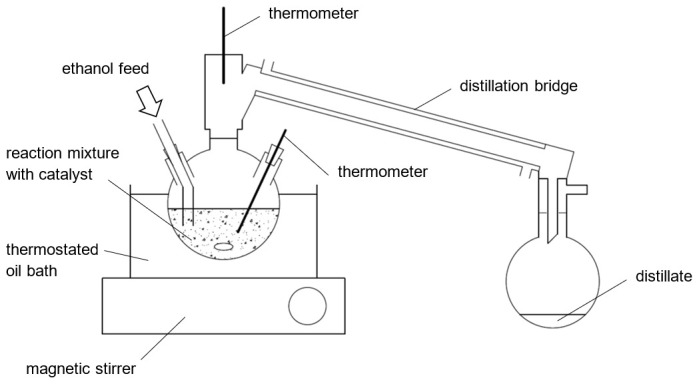
Schematic illustration of the experimental setup.

***Reactive distillation experiments.*** A conventional distillation apparatus was used to execute the reactive distillation experiments ( Figure 2). The starting mixture was prepared from highly concentrated lactic acid (100 w%) and ethanol (99.9 vol%) as in batch No. 4 ( Table 1). The reactive mixture was magnetically stirred in a 0.25 L round bottom flask. The flask was heated using a thermal oil bath. A reference sample was taken before the catalyst was added (Amberlyst 46, weight fraction w _cat_=0.1). Distillate was continuously removed by a distillation bridge and monitored by volumetric measurements. Ethanol was added manually every 10 minutes to compensate for ethanol losses into the distillate and maintain the initial molar reactant ratio of n _ethanol_/n _lactic acid_= 3:1.

The effect of ethanol feed purity and distillate rate on the reaction kinetics were studied considering two purity levels of ethanol as stripping agent (96% and 99.9%). The distillate rate was controlled by adjusting the oil bath temperature. The heat duty was determined indirectly from data of the feed and distillate flux using an energy balance. The effective specific heat duty ${\dot{\text{q}}}_{\text{eff}}$ was calculated according to Equation 1, where m _r_ is the mass of the reaction mixture, ${\dot{\text{m}}}_{\text{F}}$ is the feed flux, h _F_ is the specific enthalpy of the feed, ${\dot{\text{m}}}_{\text{D}}$ is the distillate flux and h _D_ is the specific enthalpy of the distillate.


$$\mathrm{Eq}.\;1:\;{\dot{\text{q}}}_{\text{eff}}=\frac{1}{{\text{m}}_{\text{r}}}({\dot{\text{m}}}_{\text{D}}\cdot {\text{h}}_{\text{D}}-{\dot{\text{m}}}_{\text{F}}\cdot {\text{h}}_{\text{F}})$$

***Serial setup of reaction and distillation.*** A serial setup of batch reactors and distillation steps was used as benchmark to assess simultaneous reactive distillation. Batch reaction was conducted as described above. After a certain reaction time the mixture was cooled to ambient temperature and catalyst was remove by filtration (paper filter). Ethanol and water were removed by vacuum distillation (10 kPa, 333K, Büchi R205). Mass and composition of the distillate were measured. The amount of ethanol removed was replaced by fresh ethanol as a feed. The obtained mixture was used as starting mixture for the next batch reaction step.

## Results and discussion

### Batch experiments

**Figure 3.**
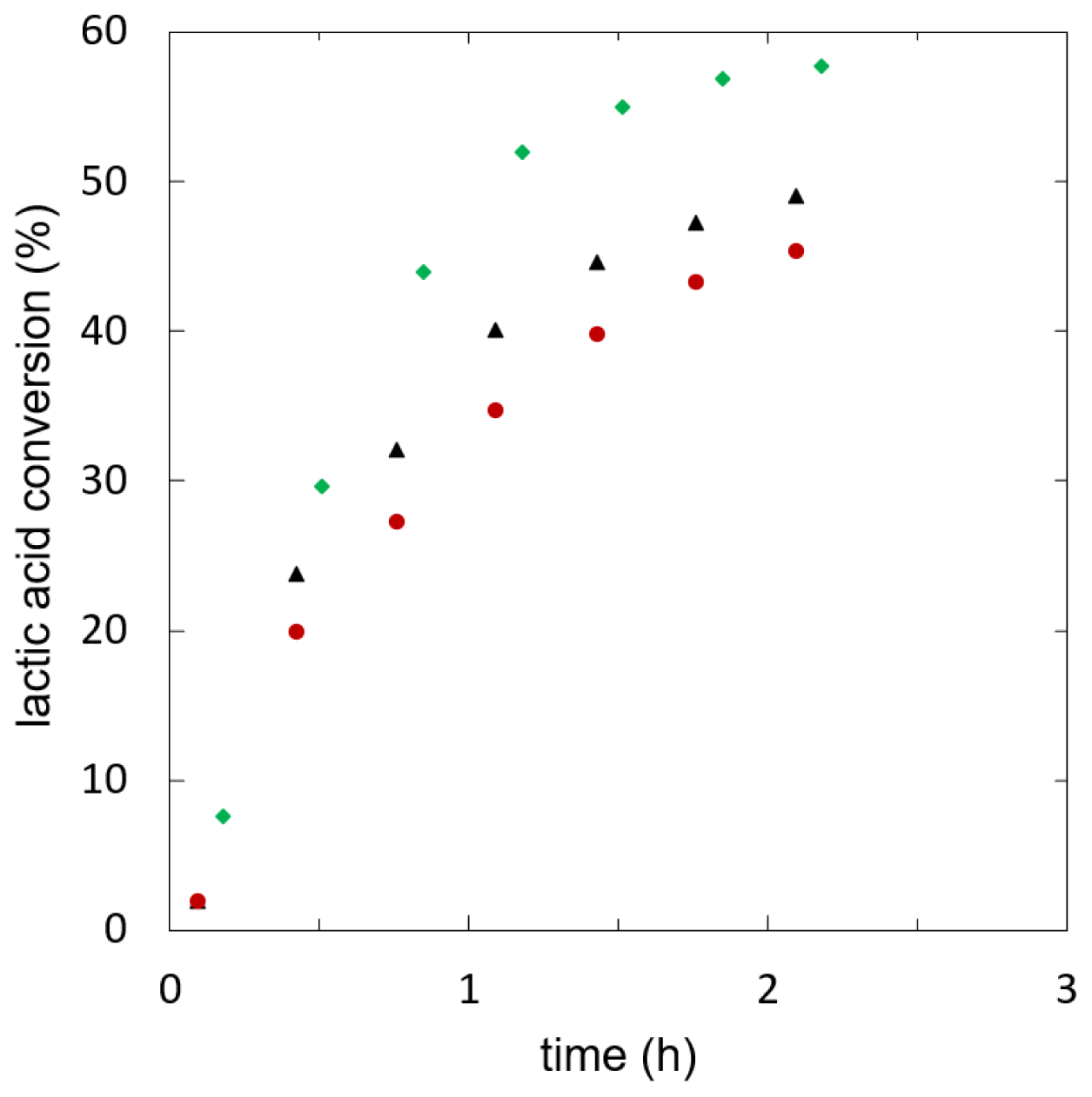
**Batch esterification of lactic acid with ethanol using different heterogeneous catalysts (w _cat_=0.1): Amberlyst 15w (** 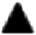 **), Amberlyst 46 (** 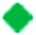 **), Amberlyst 48 (** 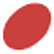 **).**

***Influence of catalyst.*** Three different catalysts were evaluated in batch experiments for the esterification of lactic acid with ethanol at a catalyst weight fraction of w _cat_=0.1 at reflux temperature (355K). The reaction kinetics are shown in Figure 3. Amberlyst 46 showed the highest catalytic activity under the studied conditions. Using this resin, 98% of the equilibrium conversion was reached within 2 h. It should be stressed that Amberlyst 46 has a lower density (600 g/L) than Amberlyst 48 (820 g/L) and Amberlyst 15 (780 g/L). Hence, the volume fraction of catalyst used in the experiments was Amberlyst 46 > Amberlyst 15 > Amberlyst 48.

***Influence of the starting mixture.*** A variation of the starting mixture regarding the initial water fraction was done in batch experiments at a catalyst weight fraction of w _cat_=0.1 (Amberlyst 46) at reflux temperature (355K). Table 2 shows the equilibrium values for all considered concentration levels.

**Table 2.** Equilibrium values for batch esterification of lactic acid with ethanol using different starting mixtures.

Initial water fraction x _w,0_ (mol/mol)	Lactic acid conversion at equilibrium X _LA,Eq_ (-)	Time to equilibrium t _Eq_ (h)
0.00	0.71	1
0.09	0.65	2
0.19	0.59	2.5
0.29	0.56	5

**Figure 4.**
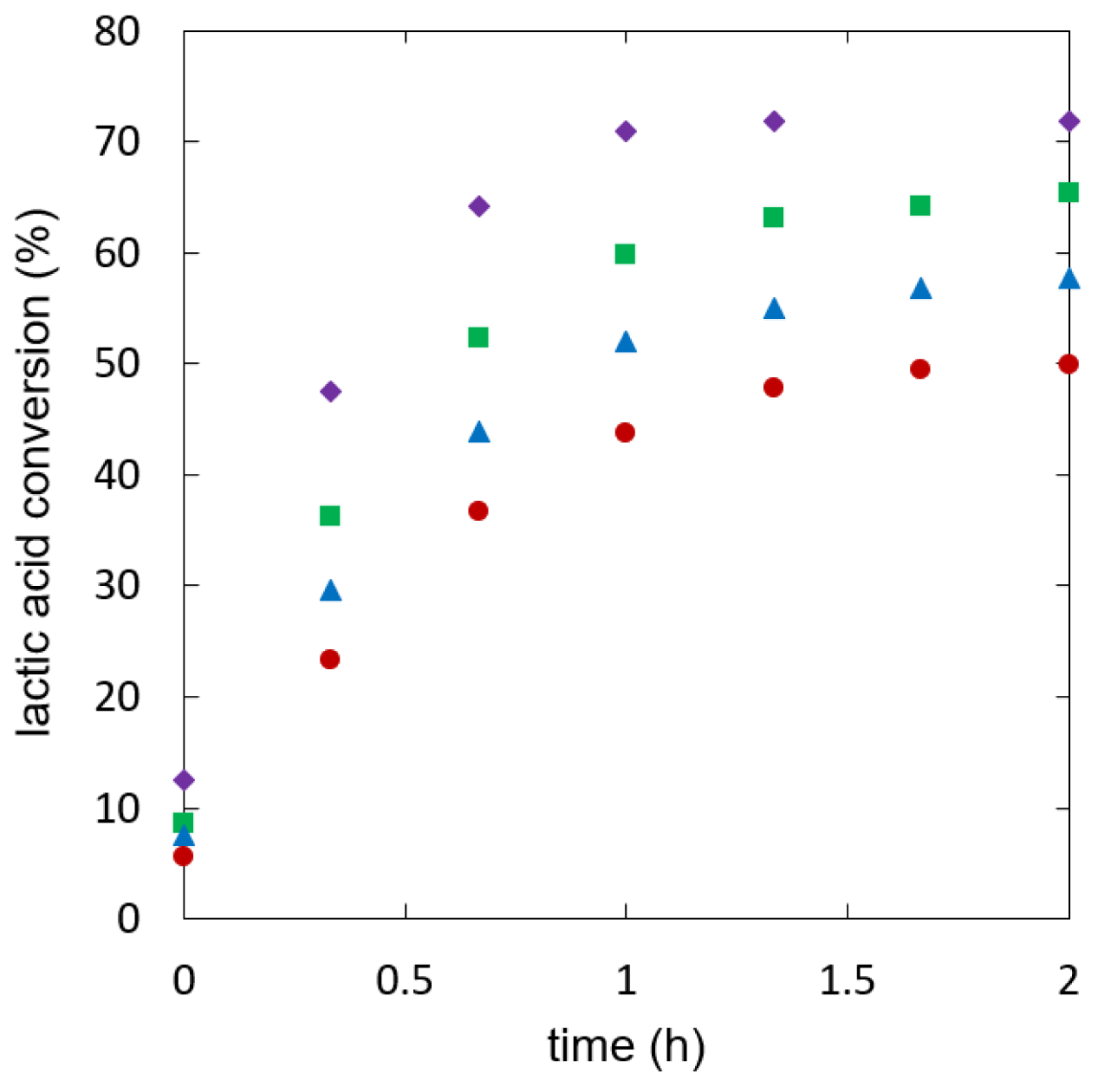
**Reaction kinetics of ethyl lactate formation in batch experiments using different starting mixtures, initial water fraction (mol/mol): 0.00 (** 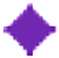 **), 0.09 (** 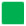 **), 0.19 (** 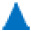 **), 0.29 (** 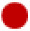 **).**

A low initial water fraction was beneficial to the equilibrium conversion as well as the reaction kinetics. When dehydrated starting mixtures were used the chemical equilibrium was reached after 1 h at 71% lactic acid conversion, compared to 59% lactic acid conversion after 2.5 h when using commercial grade chemicals (19 mol% initial water fraction). Figure 4 shows lactic acid conversion over time.

Removing water from lactic acid feed before mixing with ethanol showed notable advantages. The challenges of the reactive separation process can be significantly reduced due to very low water content of starting mixtures. When preparing the starting mixture, the earliness of mixing is of major importance. Preferably, lactic acid is mixed with ethanol immediately after dehydration. By this, the formation of lactic acid oligomers can be suppressed. Oligomerisation of lactic acid (and the hydrolysis of oligomers, respectively) proceeds relatively slow compared to ethyl lactate formation ( Pereira *et al.*, 2008). Therefore, it should be prevented in order to avoid limitations in the supply of lactic acid monomers during ethyl lactate formation.

### Reactive distillation experiments

**Figure 5.**
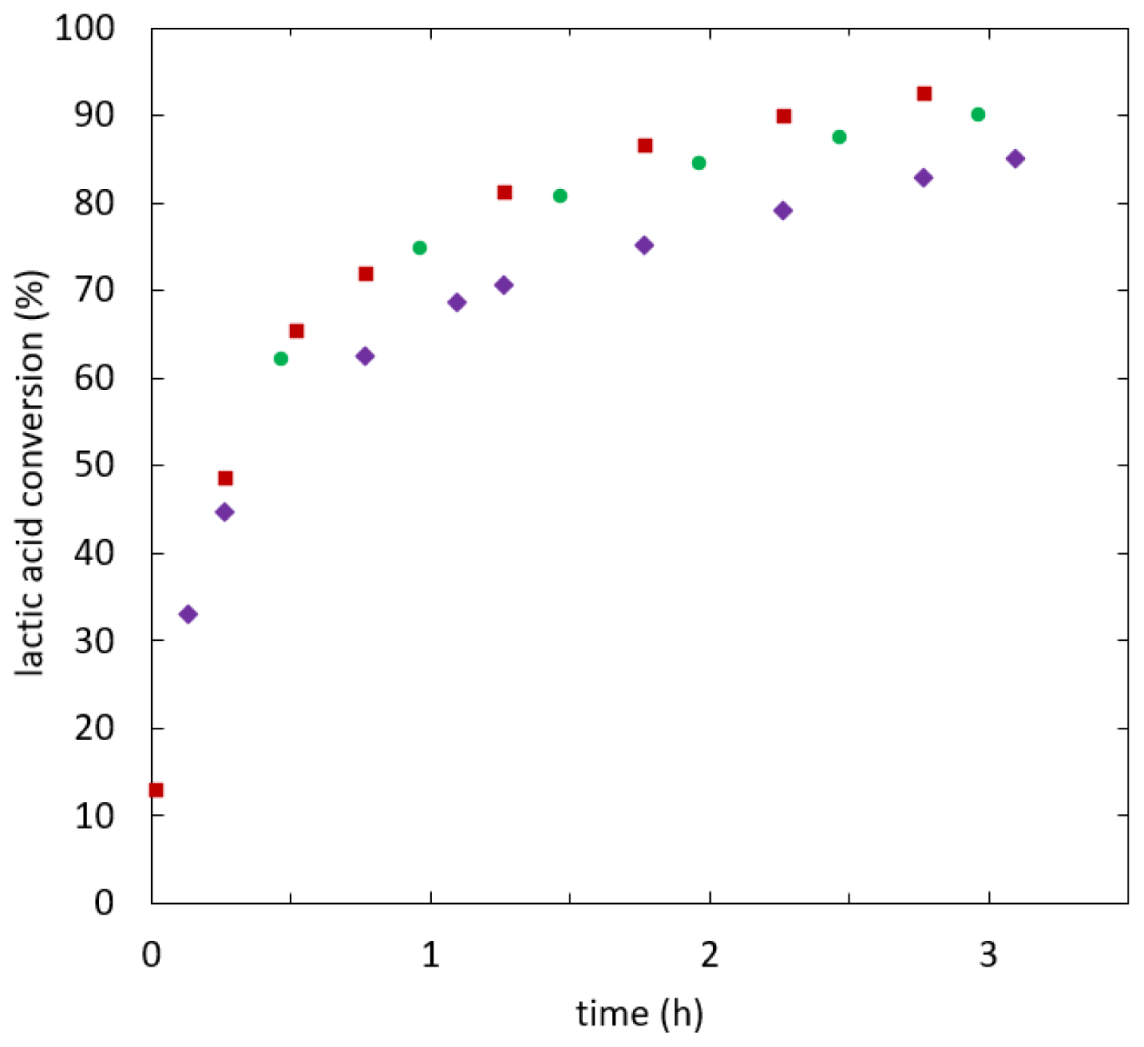
**Reaction kinetics for ethyl lactate formation in semi-batch reactive distillation, specific effective heat duty (W/kg): 112.6 (** 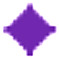 **), 121.5 (** 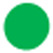 **), 141.8-209.7 (** 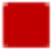 **).**

Ethanol was used as stripping agent for removing water during semi-batch reactive distillation experiments. The influence of the distillate rate and the corresponding heat duty on the reaction kinetics was evaluated. Figure 5 shows lactic acid conversion over time.

**Figure 6.**
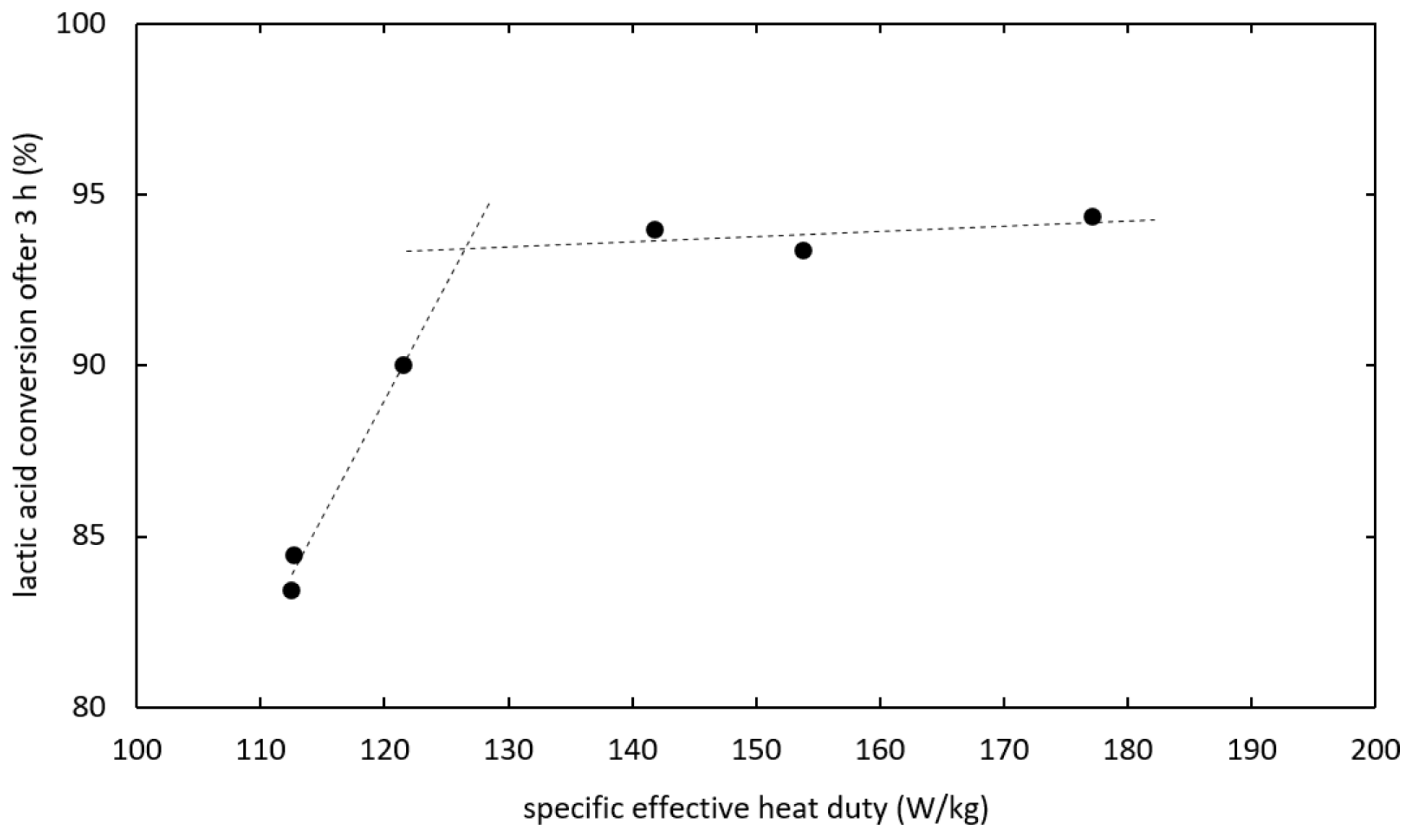
Influence of heat duty on ethyl lactate production via reactive distillation.

As shown in Figure 6, the kinetics was improved by increasing the heat duty until reaching an optimum. When the heat duty was increased further no significant change in the reaction kinetics was observed. In experiments the optimal kinetics was reached at 141.8 W/kg specific effective heat duty and 0.488 kg/kg h specific distillate rate, whereas the optimum for the heat duty is expected around 127 W/kg. The separation of water from the bulk phase is not expected to limit the reaction kinetics at optimal conditions, as increasing separation capacity did not additionally enhance the reaction.

**Figure 7.**
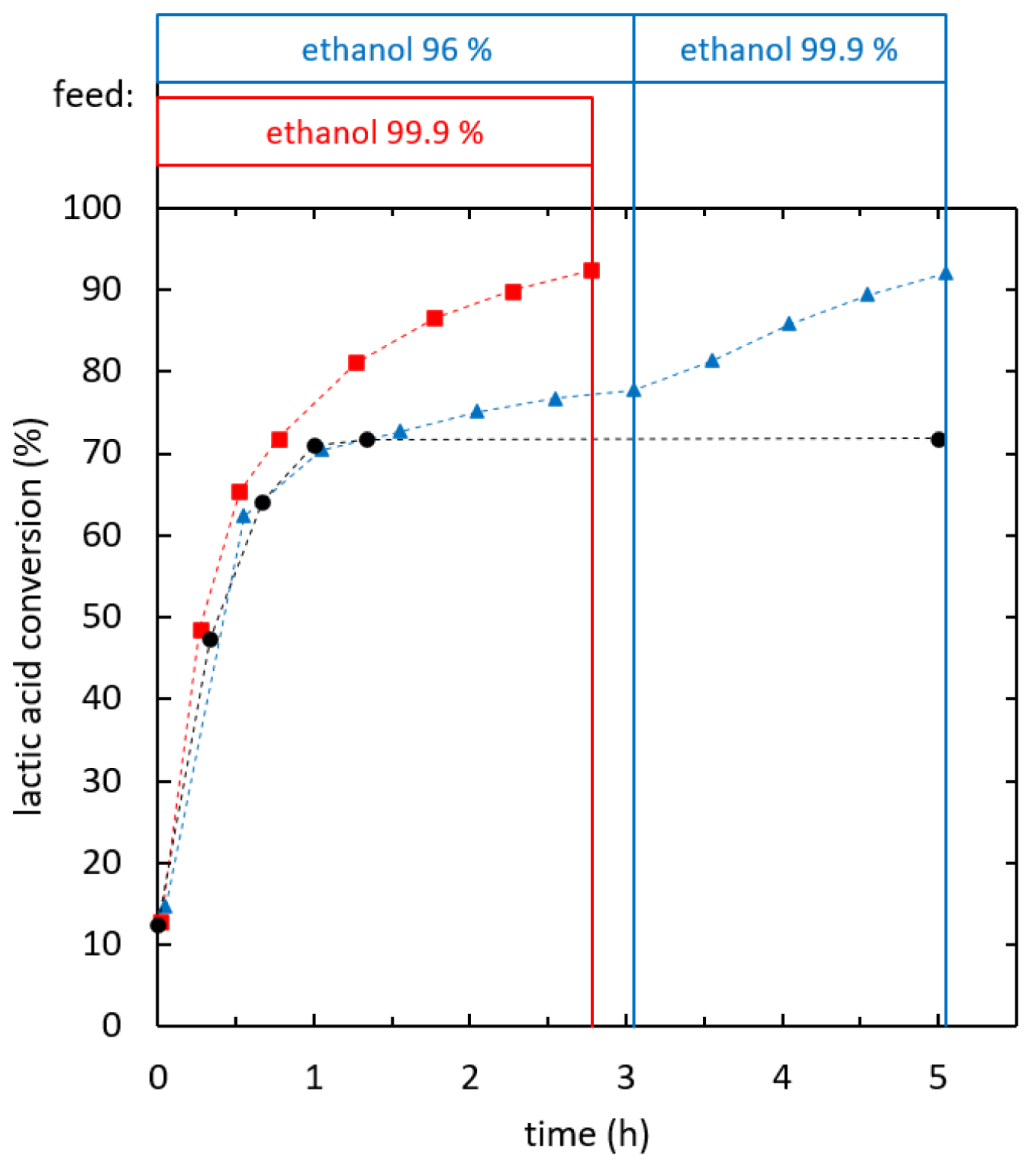
**Influence of ethanol feed purity on ethyl lactate production via reactive distillation (** 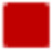 **) and (** 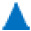 **), batch reaction (** 
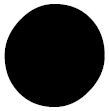
 **).**

The influence of ethanol feed purity was evaluated at a specific effective heat duty in the range of 140-155 W/kg. Figure 7 shows the reaction kinetics of ethyl lactate formation in reactive distillation experiments using ethanol of two different purity levels (96% and 99.9%) as stripping agent. When 96% ethanol was used, reactive distillation performed similarly to batch reaction, whilst the conversion was below the batch equilibrium. A conversion of 70% was reached after 1 h. Then, the reaction proceeded further and the batch equilibrium was overcome. After 3.1 h a conversion of 75% was reached. A switch of the stripping agent to dehydrated ethanol after the first reaction phase was tested, which increased the reaction rate. Using dehydrated ethanol as a stripping agent from the start lead to superior reaction kinetics even below the batch equilibrium. A conversion of 90% was reached after 2.27 h of reaction. The overall feed ratio was 4.1 kg ethanol/kg lactic acid. At this operation point the optimum of the reaction kinetics for integrated reactive distillation was found.

The obtained reaction kinetics was superior to similar processes reported in the literature, where a lactic acid conversion of about 90% was reached after 4 h ( Khunnonkwao *et al*., 2012; Tanaka *et al.*, 2002). This can be explained by the reduced water content of the feedstock used in this work.

### Serial setup of reaction and distillation

A serial setup of batch reactors and distillation units was used as a benchmark to assess the simultaneous reactive distillation under optimal conditions. In a first step the reaction was carried out until approaching batch equilibrium. A lactic acid conversion of 69% was reached after 0.83 h. Ethanol and water were almost entirely removed in a vacuum distillation step and replaced by dehydrated ethanol (99.9%). The starting mixture for the second reaction step was almost water-free again. The reaction was intensified and the chemical equilibrium was obtained after 0.5 h of reaction at 87% lactic acid conversion. After a second distillation and replacement step, 91% lactic acid conversion was reached at 1.8 h total reaction time. In the second distillation step a significant amount of ethyl lactate was detected in the distillate (<10 wt%), which was in a similar range as the additional ethyl lactate formed in reaction 3.

**Figure 8.**
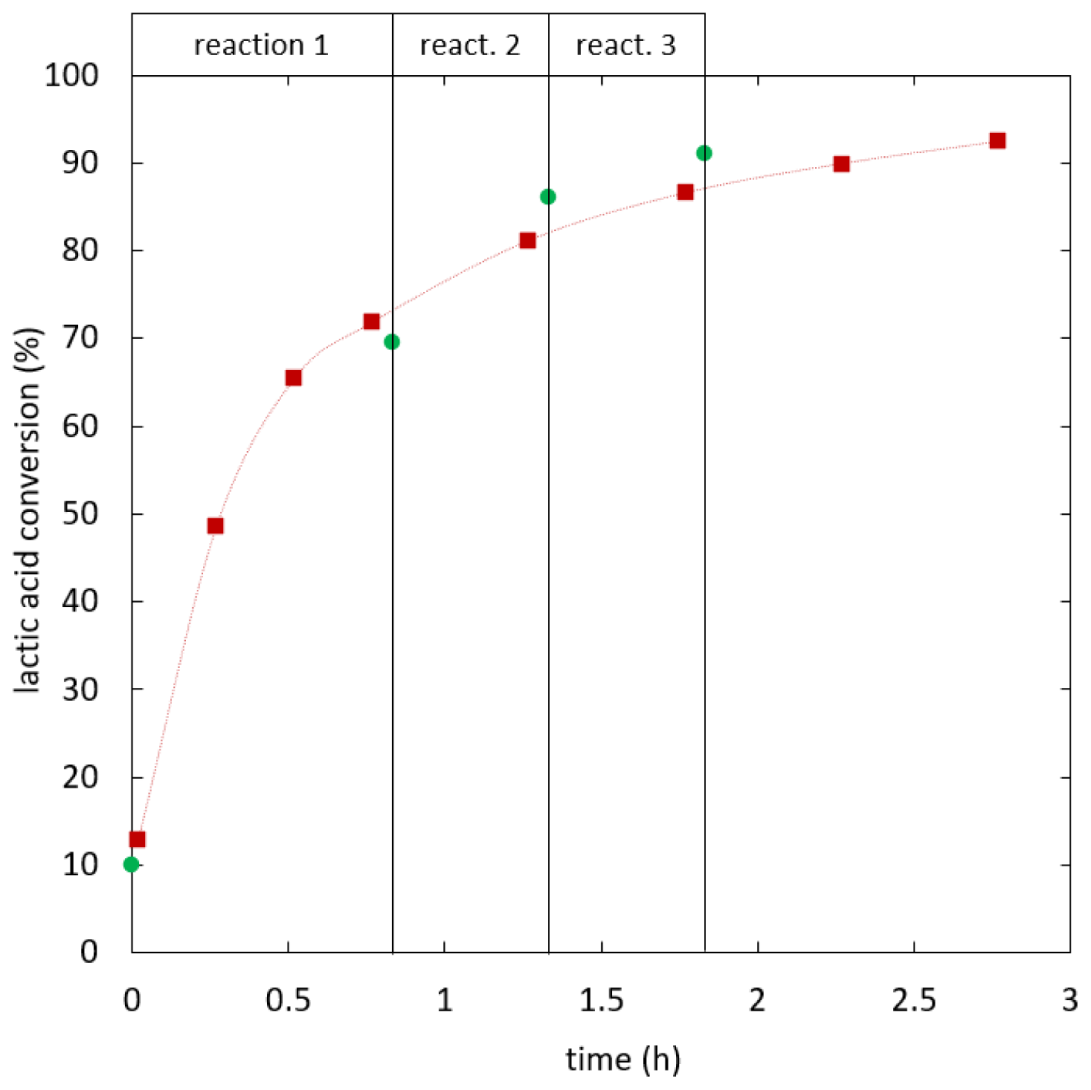
**Integrated reactive distillation (** 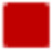 **) compared to a serial setup of batch reactions and distillation steps (** 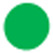 **) at 99.9% ethanol feed purity.**

In Figure 8 the series of batch reaction and distillation is compared to the simultaneous reactive distillation under optimal conditions. Both processes showed similar reaction kinetics. The serial setup is more favourable in terms of efficiency, as the overall feed ratio was 3.8 kg ethanol/kg lactic acid for 90% conversion compared to 4.1 kg/kg for the simultaneous process. If two reaction steps are considered the overall feed ratio was 2.6 kg/kg compared to 3.2 kg/kg required to reach 86% lactic acid conversion in integrated reactive distillation.

**Figure 9.**
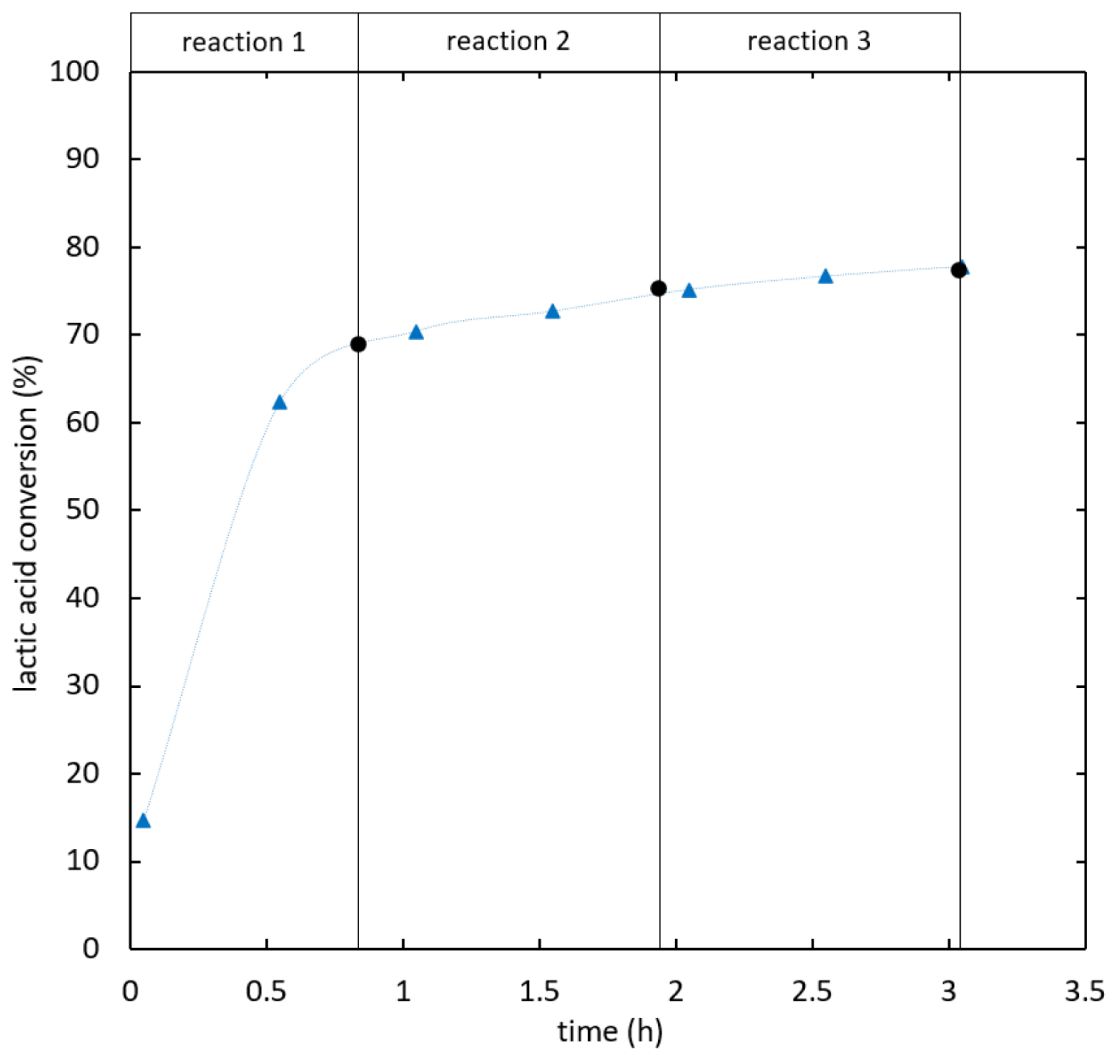
**Integrated reactive distillation (** 
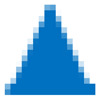
 **) compared to a serial setup of batch reactions and distillation steps (** 
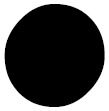
 **) at 96% ethanol feed purity.**

The influence of feed purity in the serial process was tested. The same procedure as described above was used; however, 96% ethanol was added in the replacement steps. The results were compared to combined reactive distillation with the same feed purity in Figure 9. The reaction kinetics of both processes was similar. Ethyl lactate was detected in the distillate of both intermediate distillation steps.

### Design of a continuous process

In typical reactive distillation technology the process is intensified by the simultaneous separation of products during the reaction. However, ethyl lactate production is not a straight-forward optimization task. Neither of the reaction products exhibits the highest, nor the lowest volatility of the multi-component system. In this case optimal separation is obtained without reflux, using ethanol as stripping agent for the side-product water.

Even at optimal reaction conditions the required residence time for 90% lactic acid conversion was determined to be >2 h. Reactive distillation in a vertical column would therefore require either an extensive column height, or a very low flow rate in the vertical direction that could only be attained by special column installations.

In a horizontal setup of tubular reactors, which enables simultaneous distillation, low flow rates could easily be established. However, such a setup would require a complex regulating and heating system and many feed points for ethanol. Membrane reactors would probably offer a more efficient option, if simultaneous separation was envisaged.

**Figure 10.**
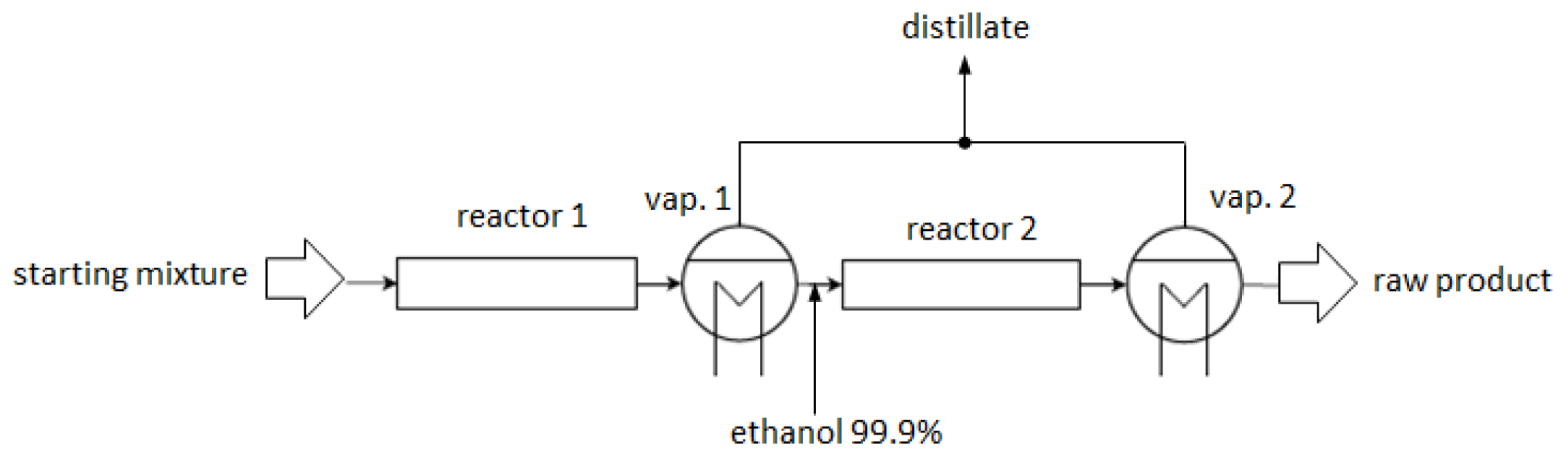
Schematic flowsheet of a continuous process for ethyl lactate production via distillation enhanced esterification of lactic acid with ethanol.

In this work a different approach to process intensification by means of distillation is proposed. The flow sheet of the continuous process is shown in Figure 10. A concept with two tubular reactors, an intermediate distillate replacement step and the downstream separation of ethanol and water offers favourable reaction kinetics and high reactant conversion while keeping losses of ethyl lactate into the distillate at a low level. A second intermediate distillation step is not recommended, because the losses of ethyl lactate into the distillate would be of similar magnitude as the additionally formed ethyl lactate in a third reaction step. The proposed setup can be built compactly, is easily scalable and offers very efficient operation.

The use of dehydrated ethanol (99.9%) is crucial to the effective separation of water. Significant process intensification could only be achieved using a dehydrated ethanol feed as stripping agent. For efficient operation of the process – especially from an economic perspective – a unit for the recovery of ethanol from the distillate is required. Dehydration of ethanol is a well-established process in industry and economically viable at a large scale. It can be done either by adsorption of water on a molar sieve, or by membrane processes such as pervaporation ( Baeyens *et al.*, 2015; Jyothi *et al.*, 2019; Vane, 2008).

## Conclusion

In this work the feedstock requirements for ethyl lactate production via reactive distillation were evaluated. Removing water from lactic acid feedstock solutions before mixing with ethanol was tested in batch experiments and applied to reactive distillation. These starting mixtures showed significant advantages that reduced the challenges of the reactive separation task. The earliness of mixing is of major importance to supress oligomerization of lactic acid. The said method for preparing starting mixtures seems especially reasonable if ethyl lactate production is integrated into a versatile biorefinery that produces both feedstocks, namely ethanol and lactic acid.

A semi-batch reactive distillation process was optimized regarding reaction kinetics and energy efficiency. A lactic acid conversion of 90% was reached after 2.3 h reaction time. The obtained reaction kinetics was superior to similar processes reported in the literature, where a lactic acid conversion of about 90% was reached after 4 h. ( Khunnonkwao *et al.*, 2012; Tanaka *et al.*, 2002) This can be explained by the reduced water content of the feedstock used in this work. It was found that dehydrated ethanol (99.9%) feedstock is necessary to establish significant process intensification. For efficient operation the recovery of ethanol from the distillate is essential. Dehydration of ethanol is a well-developed process and in many cases already established at sites that produce ethanol as a fuel additive.

Integrated reactive distillation under optimal conditions was compared to a serial setup of reactors and distillation steps. Similar process intensification at a lower overall feed ratio (3.8 kg ethanol/kg lactic acid for 90% conversion, 2.6 kg/kg for 86% conversion) was reached with the serial setup. Based on these results a continuous production process for distillation enhanced esterification was presented. Low operation costs are expected due to efficient separation of water, as well as low investment costs for compact equipment due to high space-time-yield. The process is easily scalable and could be integrated into existing or new biorefinery concepts. The flexibility of a biorefinery could be enhanced as ethyl lactate production does not require enantiomeric purity of lactic acid feedstock and could therefore compensate for quality variations of chemical building blocks.

## Data availability

### Underlying data

Zenodo: Underlying Data of "Ethyl lactate production by reactive distillation – optimization of reaction kinetics and energy efficiency". http://doi.org/10.5281/zenodo.4916874 ( Stipsitz *et al.*, 2021).

Data are available under the terms of the Creative Commons Attribution 4.0 International license (CC-BY 4.0).
