# Supplementary material for: Ethyl lactate production by reactive distillation – optimization of reaction kinetics and energy efficiency
Source: Open Res Eur. 2021 Sep 23;1:82. Originally published 2021 Jul 20. [Version 2] doi: 10.12688/openreseurope.13744.2 (PMC10445869; doi:10.12688/openreseurope.13744.2)
Supplement: Peer review history

# Peer review: Reviewer response for version 2

**DOI:** 10.21956/openreseurope.15118.r27669

**Published:** 2021-09-30

**Authors:** Juan Gabriel Segovia-Hernandez

**Affiliations:** 1 Chemical Engineering Department, Campus Guanajuato, Universidad de Guanajuato, Guanajuato, Mexico

Once the authors have addressed the comments of the reviewers, the paper has improved and it can be accepted for indexing.

Is the study design appropriate and does the work have academic merit?

Yes

Is the work clearly and accurately presented and does it cite the current literature?

Partly

If applicable, is the statistical analysis and its interpretation appropriate?

No

Are all the source data underlying the results available to ensure full reproducibility?

Partly

Are the conclusions drawn adequately supported by the results?

Partly

Are sufficient details of methods and analysis provided to allow replication by others?

Partly

Reviewer Expertise:

process intensification, control, optimization

I confirm that I have read this submission and believe that I have an appropriate level of expertise to confirm that it is of an acceptable scientific standard.

# Peer review: Reviewer response for version 2

**DOI:** 10.21956/openreseurope.15118.r27670

**Published:** 2021-09-30

**Authors:** María Teresa Sanz

**Affiliations:** 1 Department of Biotechnology and Food Science, University of Burgos, Burgos, Spain

I have no further comments to add. Authors have answered the proposed questions and I think the topic is of interest to be indexed.

Is the study design appropriate and does the work have academic merit?

Yes

Is the work clearly and accurately presented and does it cite the current literature?

Yes

If applicable, is the statistical analysis and its interpretation appropriate?

Not applicable

Are all the source data underlying the results available to ensure full reproducibility?

Partly

Are the conclusions drawn adequately supported by the results?

Partly

Are sufficient details of methods and analysis provided to allow replication by others?

Partly

Reviewer Expertise:

NA

I confirm that I have read this submission and believe that I have an appropriate level of expertise to confirm that it is of an acceptable scientific standard.

# Peer review: Reviewer response for version 1

**DOI:** 10.21956/openreseurope.14821.r27277

**Published:** 2021-08-02

**Authors:** María Teresa Sanz

**Affiliations:** 1 Department of Biotechnology and Food Science, University of Burgos, Burgos, Spain

The authors propose a new configuration to produce ethyl lactate from the esterification reaction of lactic acid with ethanol by combining different steps of reaction and subsequent removal of ethanol and water. The content of water was proved as an important parameter to determine the lactic acid conversion. The topic is of interest due to the properties of ethyl lactate as solvent. However, some issues could be better explained.

 The authors presented a good introduction section regarding previous studies and describing the presence of oligomers by using commercial lactic acid of 88 wt %. However, during the development of the manuscript no results were presented about the presence of oligomers, that probably would be concentrate in the ethyl lactate rich stream. Results are only reported as lactic acid conversion but a characterization in terms of chemical composition of the different streams is missing for the three experiments carried out by the authors: batch experiments, reactive distillation experiments and serial set up of reaction and distillation.

 Please, specify the analytical procedure to check the final content of water (0.00 mol/mol) for pre-treated lactic acid.

 When using the 100 (pre-treated) lactic acid the authors stated that when mixing with ethanol the formation of oligomers is suppressed. But 88 wt% commercial lactic acid has already important amounts of oligomers that would be concentrated after water removal.

 Please, in equation 1, indicate how the specific enthalpy of the feed and distillate was evaluated.

 In Figure 3, the effect of the type of catalyst is plotted. It seems that the initial reaction rate is quite similar for all the three catalyst studied, but the equilibrium conversion seems to be different. To assess this effect, longer kinetic reaction could have been carried out. Could the authors explain the effect of catalyst on equilibrium conversion?

 Please, in Table 2, include the initial reaction rate since according to the authors “a low initial water fraction was beneficial to the equilibrium conversion as well as the reaction kinetics”. The values of r _o_ could help in the description of the results.

 The authors proposed a combined intensified process based on different steps of reaction and separation of water/ethanol and then a subsequent reaction step by replacing the removed ethanol. It would have been very interested for the work to report the exact composition of the different streams plotted in Figure 10. According to the manuscript, lactic acid, ethanol and ethyl lactate were quantified by HPLC (and water and ethanol by density measurements), but no results were presented. Did the authors observe any difference between ethanol composition by HPLC and density measurements?

 A final comparison of different intensification process such as esterification process combined with pervaporation to remove water by using hydrophilic membranes would have been useful.

Is the study design appropriate and does the work have academic merit?

Yes

Is the work clearly and accurately presented and does it cite the current literature?

Yes

If applicable, is the statistical analysis and its interpretation appropriate?

Not applicable

Are all the source data underlying the results available to ensure full reproducibility?

Partly

Are the conclusions drawn adequately supported by the results?

Partly

Are sufficient details of methods and analysis provided to allow replication by others?

Partly

Reviewer Expertise:

process intensification, biomass valorization, pressurized fluids

I confirm that I have read this submission and believe that I have an appropriate level of expertise to confirm that it is of an acceptable scientific standard, however I have significant reservations, as outlined above.

# Response

**Published:** 2021-09-24

**Authors:** Peter Stipsitz

**Affiliations:** tbw research GmbH, Austria

Dear Dr. Sanz, thank you very much for the constructive review of our work. We really appreciate your suggestions for improvement. In the following paragraphs we address your individual comments.

*Please, specify the analytical procedure to check the final content of water (0.00 mol/mol) for pre-treated lactic acid.”* The final content of water was determined by a mass balance. The distillate was checked for its lactic acid content, which was <5 w% in all experiments. *“When using the 100 (pre-treated) lactic acid the authors stated that when mixing with ethanol the formation of oligomers is suppressed. But 88 wt% commercial lactic acid has already important amounts of oligomers that would be concentrated after water removal. (…)* *It would have been very interesting for the work to report the exact composition of the different streams plotted in Figure 10.”*

 It is correct, that the oligomers of commercial lactic acid were concentrated in the experiments presented in version 1. It is expected that the oligomers remained unavailable for ethyl lactate formation. The lactic acid conversion was calculated from the change of the lactic acid monomer concentration. In version 2 we present the mass balances of the continuous process and the composition of the individual streams, based on new experimental data. For these experiments hydrolyzed lactic acid feedstock was prepared using an adapted experimental procedure.

*“Please, in equation 1, indicate how the specific enthalpy of the feed and distillate was evaluated.”*

 The specific enthalpy of the feed and distillate was calculated according to its composition from values of the individual components. References to the literature values were added to the manuscript, which are also listed in the underlying data file. All measured values and calculations for each specific experiment can be found in the underlying data.

*“In Figure 3, the effect of the type of catalyst is plotted. It seems that the initial reaction rate is quite similar for all the three catalyst studied, but the equilibrium conversion seems to be different. To assess this effect, longer kinetic reaction could have been carried out. Could the authors explain the effect of catalyst on equilibrium conversion? (…)* *Please, in Table 2, include the initial reaction rate since according to the authors “a low initial water fraction was beneficial to the equilibrium conversion as well as the reaction kinetics”. The values of r _o_ could help in the description of the results.”*

 We listed the initial reaction rates r _0_ in Table 3 and the additional Table 2 to increase comparability of the kinetics results. The equilibrium conversion was independent of the type of catalyst. After 24 h of reaction the same equilibrium conversion was reached with all three catalysts. Within the time shown in Figure 3 the equilibrium was not yet reached. Equilibrium values are presented in Table 3.

# Peer review: Reviewer response for version 1

**DOI:** 10.21956/openreseurope.14821.r27279

**Published:** 2021-07-22

**Authors:** Juan Gabriel Segovia-Hernandez

**Affiliations:** 1 Chemical Engineering Department, Campus Guanajuato, Universidad de Guanajuato, Guanajuato, Mexico

Several details must be clarified before the paper is ready for indexing.

1. The authors should explain in detail how they carried out each of the experiments. There are a lot of information loss that would not allow anyone interested to reproduce the experiments shown in the paper. Authors need to be more explicit in the execution of the experiments and give more detail to be reproduced.
2. It is necessary to conduct a more detailed statistical study of the results. It is necessary to validate the experiments and their reliability. On the other hand, it is necessary to carry out a detailed statistical design of experiments to correlate all the variables analyzed to observe the effect of all of them and which of them have more or less impact on the case study. This information is lost in the document.
3. The results are of interest. But the authors need to establish an in-depth analysis to correlate the information obtained and to establish a firm conclusion from the results. The analysis is very superficial.
4. If it is not the goal of the work, but the authors should assess the economic  impact of the proposal. Is it economically viable the proposal submitted by use in industry, for example?.
5. Authors should clearly establish which is the novelty of the work. What is really new in this paper? What is the difference with other previously published works on the subject?

Is the study design appropriate and does the work have academic merit?

Yes

Is the work clearly and accurately presented and does it cite the current literature?

Partly

If applicable, is the statistical analysis and its interpretation appropriate?

No

Are all the source data underlying the results available to ensure full reproducibility?

Partly

Are the conclusions drawn adequately supported by the results?

Partly

Are sufficient details of methods and analysis provided to allow replication by others?

Partly

Reviewer Expertise:

process intensification, control, optimization

I confirm that I have read this submission and believe that I have an appropriate level of expertise to confirm that it is of an acceptable scientific standard, however I have significant reservations, as outlined above.

# Response

**Published:** 2021-07-26

**Authors:** Peter Stipsitz

**Affiliations:** tbw research GmbH, Austria

Thank you very much for your review report. We appreciate your suggestions and will soon provide a new version of the manuscript, that addresses all of your comments.

# Response

**Published:** 2021-09-24

**Authors:** Peter Stipsitz

**Affiliations:** tbw research GmbH, Austria

Dear Dr. Segovia-Hernandez, thank you very much for the constructive review of our work. We really appreciate your suggestions for improvement. In the following paragraphs we address your individual comments.

1. *“The authors should explain in detail how they carried out each of the experiments. There are a lot of information loss that would not allow anyone interested to reproduce the experiments shown in the paper. Authors need to be more explicit in the execution of the experiments and give more detail to be reproduced.”*
  In version 2 we described the methods and validation of the experiments more precisely.
2. *“It is necessary to conduct a more detailed statistical study of the results. It is necessary to validate the experiments and their reliability. On the other hand, it is necessary to carry out a detailed statistical design of experiments to correlate all the variables analyzed to observe the effect of all of them and which of them have more or less impact on the case study. This information is lost in the document.”*
  In version 2 we described the validation of the experiments more precisely. All experiments were conducted at least in duplicate, the main findings were conducted at least in triplicate and are represented by means and standard deviations in the whole document. The concept of the experimental design was explained in an additional paragraph.
3. *“* *The results are of interest. But the authors need to establish an in-depth analysis to correlate the information obtained and to establish a firm conclusion from the results. The analysis is very superficial.”*  
  We elaborated the comparison of the different experimental setups and established a more precise conclusion. The optimal process for ethyl lactate production by distillation enhanced esterification was found.
4. *“* *If it is not the goal of the work, but the authors should assess the economic impact of the proposal. Is it economically viable the proposal submitted by use in industry, for example?”* 
  Unfortunately, there is a lack of publicly available data regarding the energy demand and operation costs of the conventional process used in industry. Therefore, we compared two different setups for the process using experimental data. A simple serial setup for distillation enhanced esterification was used as a reference for assessing the integrated process. In the conclusion we added suggestions for the application of the proposed technology in integrated biorefineries.
5. *“Authors should clearly establish which is the novelty of the work. What is really new in this paper? What is the difference with other previously published works on the subject?”*
  In the introduction we described the concept and novelty of the work more precisely. The goal was to find the optimal process for ethyl lactate production by distillation enhanced esterification by comparing different experimental setups.
